# Debridement, Antibiotics, and Implant Retention (DAIR) in Acute Hematogenous Total Knee Arthroplasty Infections: A Case Series

**DOI:** 10.7759/cureus.69497

**Published:** 2024-09-16

**Authors:** Muhammad Nadzim Abdull Sitar, Muhammad Fathi Hayyun, Juzaily F Leong, Rizal Abdul Rani, Nor Hamdan Mohamad Yahaya

**Affiliations:** 1 Orthopedics and Traumatology, Universiti Kebangsaan Malaysia, Kuala Lumpur, MYS

**Keywords:** dair, debridement and implant retention, gentamycin, infected total knee arthroplasty, prosthetic joint infection, stimulant

## Abstract

Prosthetic joint infection (PJI) is a catastrophic complication in total knee arthroplasty (TKA). Implant retention with eradication of infection to maintain limb function is ideal and debridement, antibiotics, and implant retention (DAIR) have been reported to have variable success rates depending on several factors, including the duration of infection, the host immunity, the virulence of the causative microorganism, and the technique employed. In this case series, we present a series of successful cases presented with PJI after TKA and were treated using DAIR either via impregnation of vancomycin beads or calcium sulfate beads (antibiotic stimulan).

## Introduction

Infection is one of the most severe complications and a common reason for revision, along with aseptic loosening and instability, following total knee arthroplasty (TKA). It is estimated that the incidence of this condition ranges from 1% to 2% following primary prosthesis placement and can reach up to 5-6% during revision surgery [1]. The most effective method for treating chronic infection with implant loosening is two-stage reimplantation, as these infections are difficult to manage with debridement and irrigation with prosthetic retention, followed by antibiotic therapy (DAIR) [2]. DAIR is one of the preferred treatments for acute postoperative and acute hematogenous infections of TKA with less than four weeks duration of symptoms. Nevertheless, the success rate of this treatment is subject to significant variability in the literature, ranging from 18% to 100% [3]. This variability is influenced by the patient's comorbidity, the duration of the infection, the microorganisms involved, the debridement technique, the type of antibiotic, and the duration of antibiotic therapy. There is ongoing debate in the current literature regarding the appropriateness of performing DAIR for both acute postoperative and acute hematogenous infections. This is due to the fact that a significant number of patients with acute hematogenous infections ultimately experience a relapse of infection following this procedure and about 42% of hematogenous prosthetic joint infection (PJI) the origin remains unknown [4]. In this report, we present a series of successful cases of knee PJI that were treated with DAIR and the insertion of vancomycin beads or calcium sulphate beads (antibiotic stimulan).

## Case presentation

Case 1

**Figure 1:**
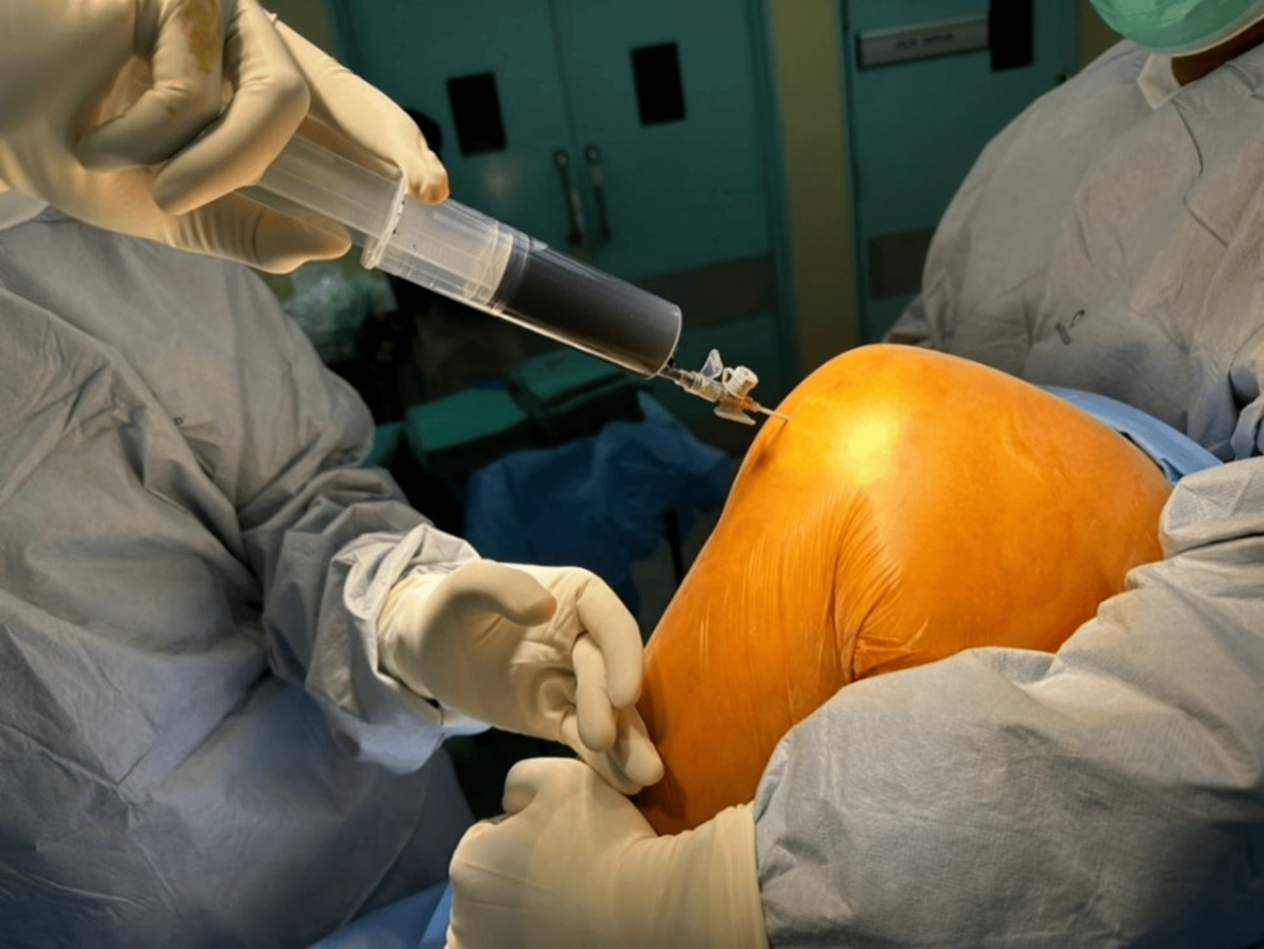
Methylene blue injection prior to incision

**Figure 2:**
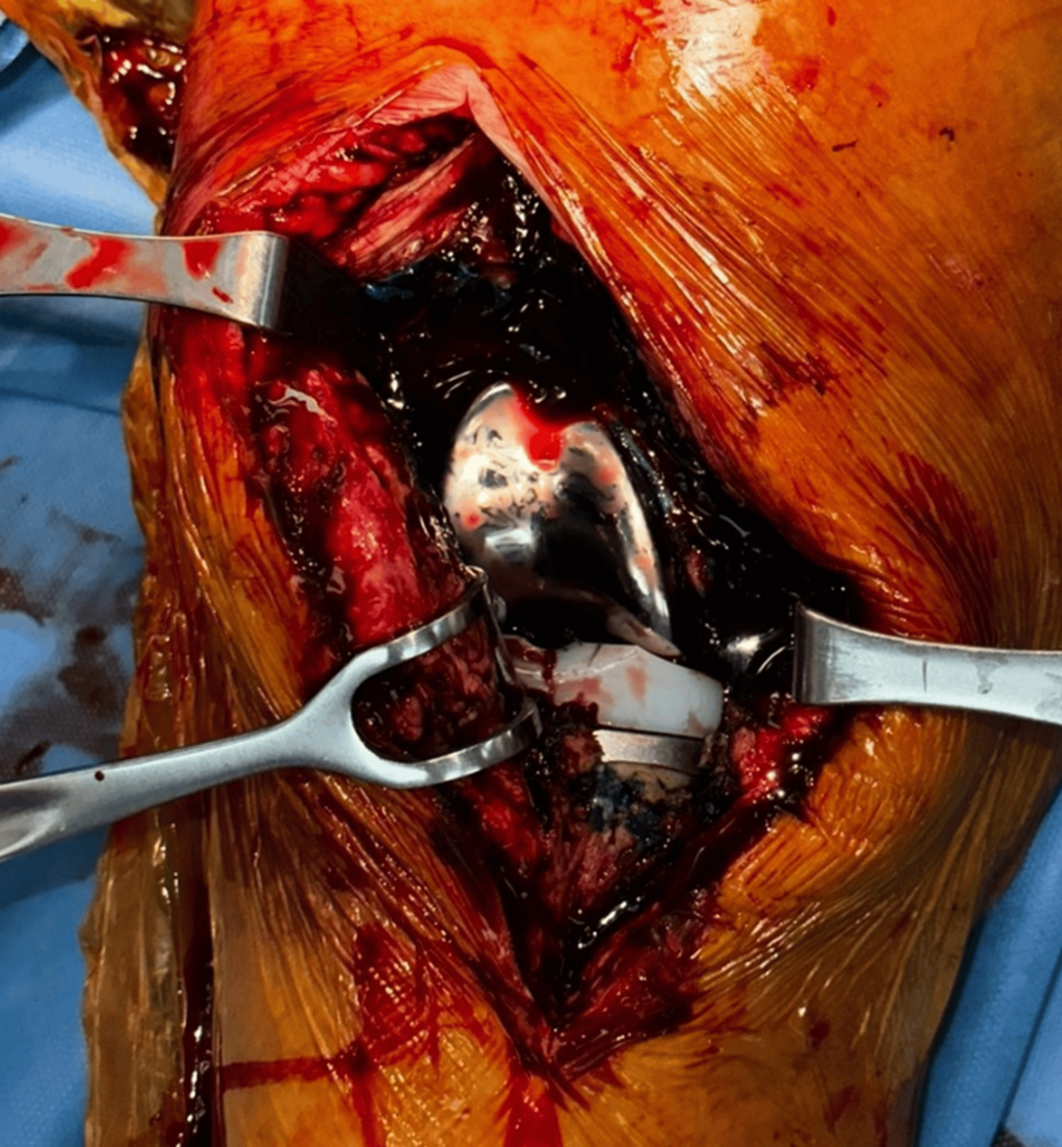
Staining of tissues with methylene blue

**Figure 3:**
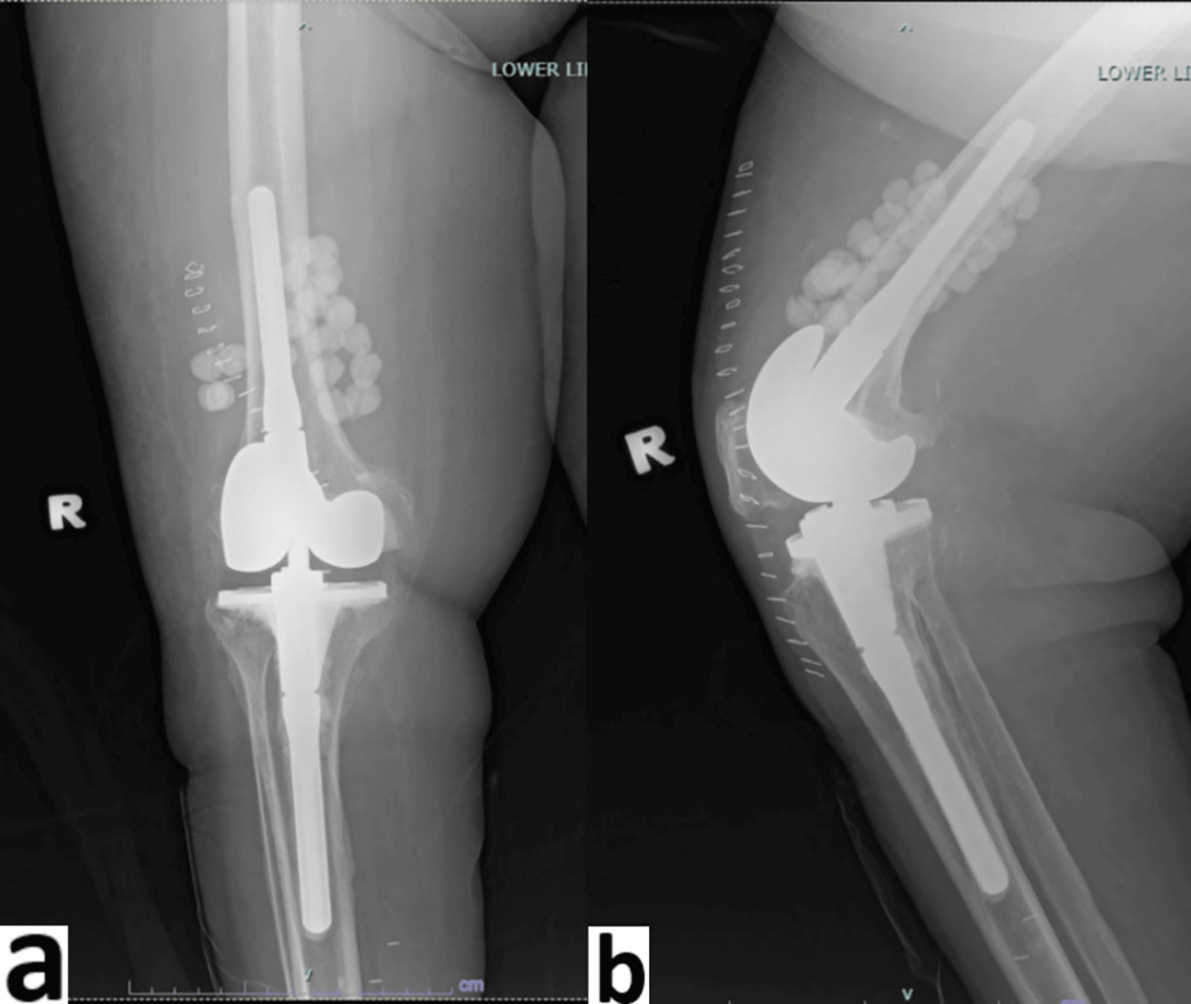
Post-operative radiograph of right knee with vancomycin beads insertion a) anteroposterior view; b) lateral view

A 70-year-old lady with a history of TKA done 10 years ago presented to us with a complaint of right knee pain and swelling for eight days, diagnosed as right knee PJI with Tsukayama stage 3 [2], acute hematogenous infection. Her blood culture and sensitivity were positive for *Staphylococcus aureus*. The patient underwent debridement, antibiotics, and implant retention (DAIR) with a change of polyethylene (PE) and vancomycin beads insertion the next day of diagnosis. Intraoperatively, an injection of 50 ml of 0.1% methylene blue dye into the right knee joint was done for the aid debridement (Figure 1). Upon exploration, a methylene blue stain was noted over the suprapatellar pouch extending to the posteromedial of the knee joint (Figure 2). Femoral and tibia components are well fixed, with no loosening of the implant. The tibial PE was then changed, and 4 grams of vancomycin beads with a size of about 0.5 cm × 1 cm each were packed in the suprapatellar pouch and later removed after two weeks (Figure 3). The patient was then kept in the ward for four weeks. Intraoperative tissue that was sent for culture and sensitivity was negative for the organism, and the patient was given IV Cefazolin for two weeks and then changed to Tab Rifampicin and Tab Ciprofloxacin to complete a total of 10 weeks. The septic parameters of the patient began to decrease in trend with the C-reactive protein (CRP) reduced from 28.1 mg/dL to 0.2 mg/dL (normal range less than 0.3 mg/dL) (Table 1). We followed up with the patient for six months and through this period, the midline knee wound has healed and the inflammatory markers normalized.

**Table 1:** C-reactive protein (CRP) and White blood cell (WBC) trend

Date	CRP (<0.5 mg/dL)	WBC (4-10x10^9/L)
7/4/2022	28.1	15.6
15/4/2022	13.2	12.7
23/4/2022	4.8	5.1
2/5/2022	5.7	9.9
15/5/2022	1.1	11.2
12/6/2022	0.5	8.4
20/7/2022	0.2	7.3

Case 2

**Figure 4:**
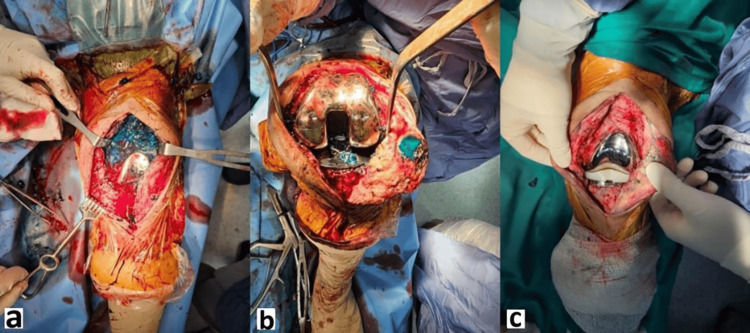
Intraoperative pictures of wound debridement a) usage of methylene blue to aid in debridement; b) removal of tibial insert; c) replacement of the tibial insert

**Figure 5:**
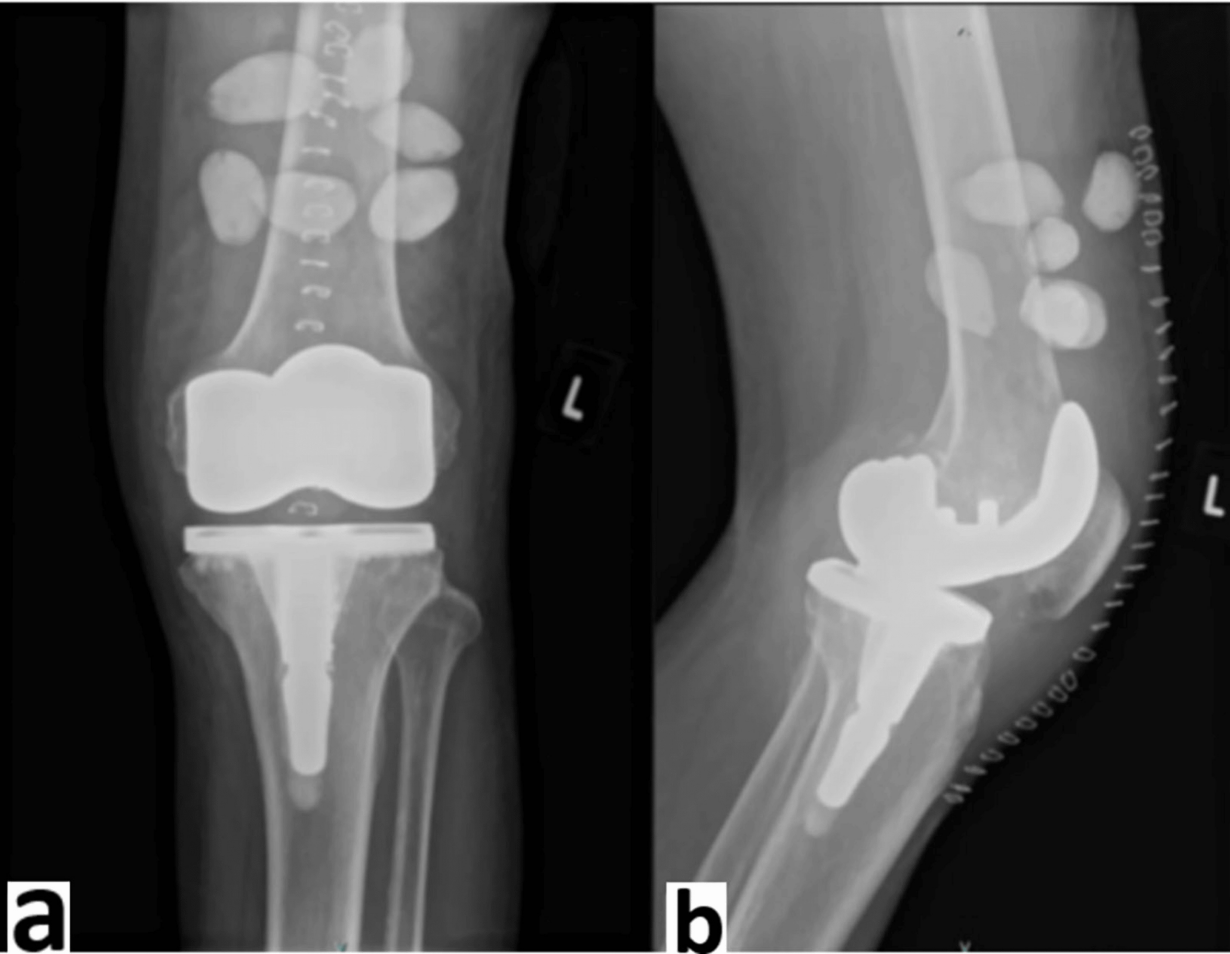
Radiographs of left knee post insertion of vancomycin beads a) anteroposterior view; b) lateral view

A 64-year-old male presented to us with acute onset of left knee pain for two days with a history of TKR done four months prior on March 18, 2022. On examination, there was swelling and tenderness over the left knee and was unable to flex his knee due to this pain. The patient was diagnosed with left knee PJI, and subsequently, the patient underwent left knee DAIR with the change of PE on the following day of diagnosis. Methylene blue was used intraoperatively to see the extent of infection (Figure 4). Intraoperative tissue culture analysis revealed the presence of Methicillin-Resistant *S. aureus* (MRSA) infection, prompting the initiation of intravenous vancomycin therapy based on the sensitivity. Following the surgery, complications arose where there was a wound breakdown at the surgical site on day 7 postoperatively, which required another debridement and replacement of the tibial insert. We decided to insert bone cement beads impregnated with vancomycin. There were six vancomycin beads with sizes about 2 cm x 2 cm each; five were positioned within the knee capsule, and another was extra-capsular and these antibiotic-impregnated beads were removed two weeks later. Intraoperative culture results following this procedure showed an absence of bacterial presence. Postoperatively, the patient regained the ability to stand with the assistance of a knee brace, maintaining the knee in an extended position, and plain radiographs were taken (Figure 5). The septic parameters of the patient began to decrease in trend with the CRP reduced from 19.67 mg/dL to 0.63mg/dL (normal range less than 0.3 mg/dL) (Table 2). The patient had completed four weeks of intravenous vancomycin and subsequently switched to oral rifampicin and fusidic acid. We follow up with patients for the next six months, and clinically, the patient shows significant improvement and also blood parameters. The inflammatory markers normalized, and the patient was able to ambulate without an assistant.

**Table 2:** C- reactive protein (CRP) and white blood cell (WBC) trend

Date	CRP (<0.5 mg/dL)	WBC (4-10x10^9/L)
23/7/2022	19.6	18.8
30/7/2022	28.7	19.7
7/8/2022	5.1	6.4
28/8/2022	1.7	8.3
4/9/2022	0.3	5.5
19/9/2022	0.6	6.4

Case 3

**Figure 6:**
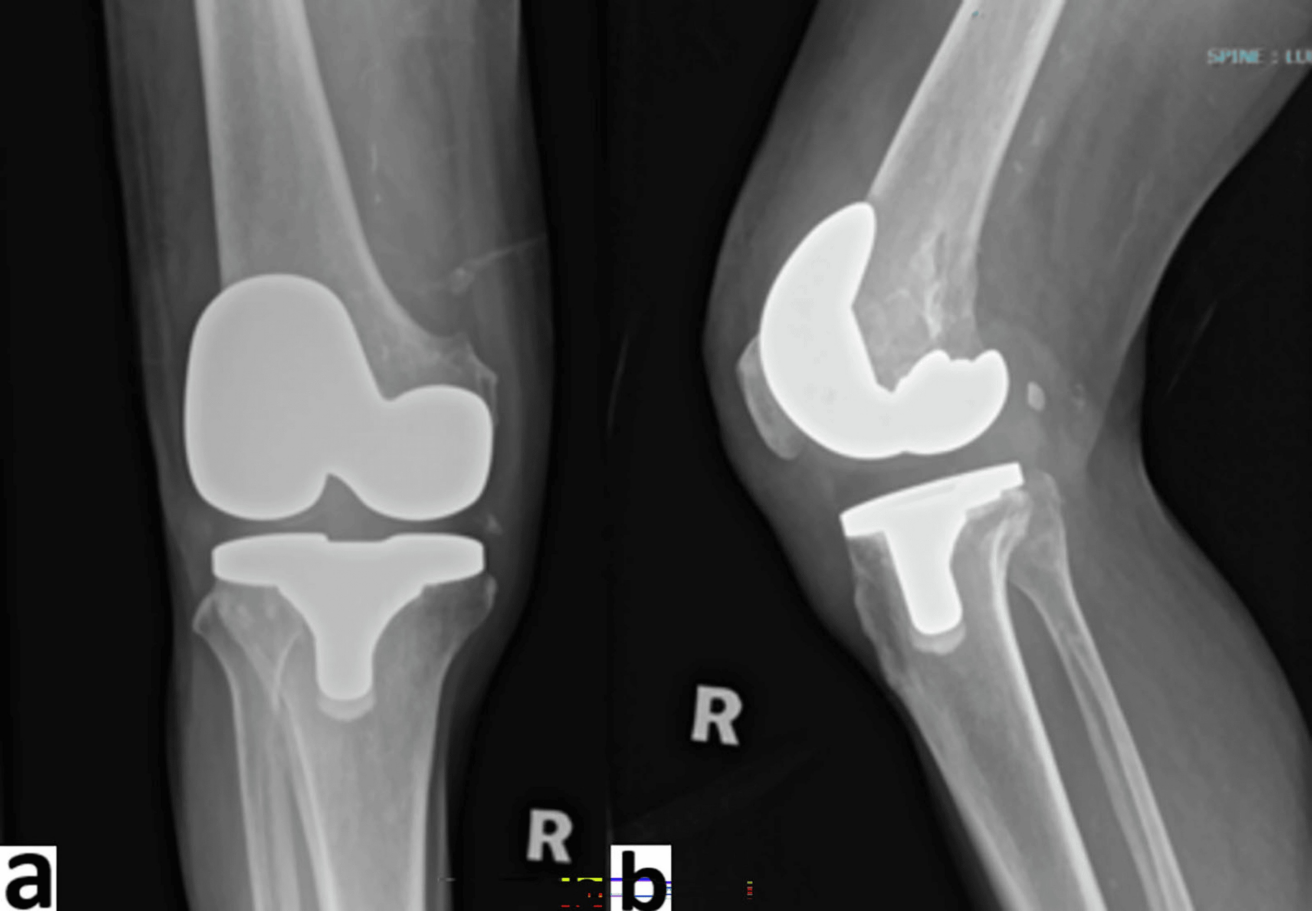
Radiographs of right knee a) anteroposterior view; b) lateral view

**Figure 7:**
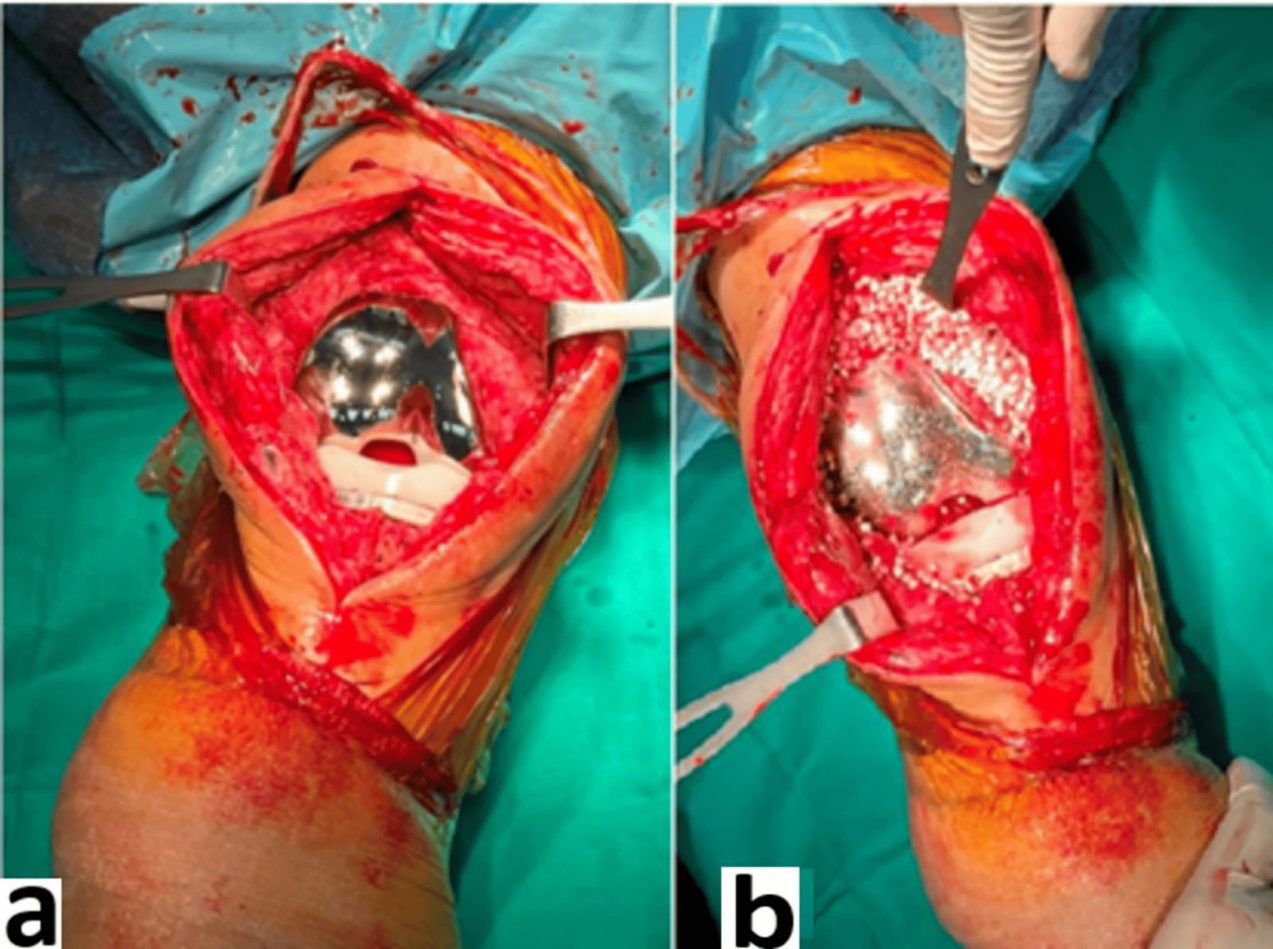
Intraoperative debridement pictures a) post-debridement wound prior to vancomycin-bone stimulan insertion; b) vancomycin-bone stimulan insertion

**Figure 8:**
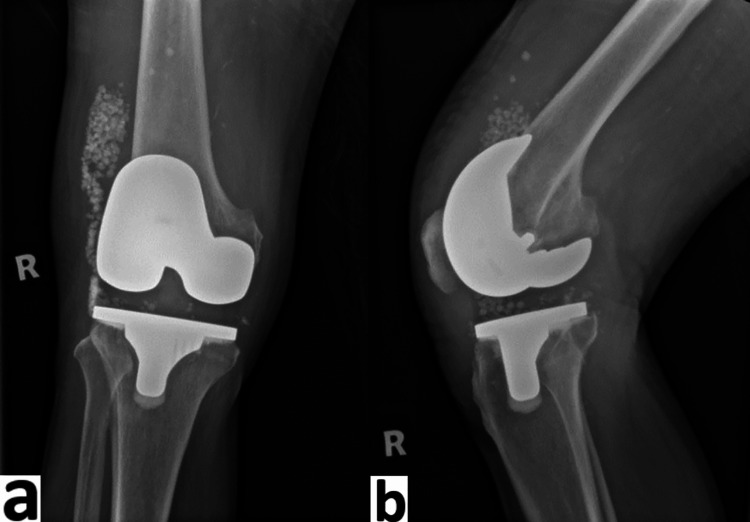
Radiograph of right knee postoperatively a) anteroposterior view; b) lateral view

A 70-year-old man with a history of right knee TKA done seven years ago presented with pain and swelling in his right knee for six days following a road traffic accident. Upon presentation, the right knee was erythematous, warm, and painful with positive effusion. The range of motion of the right knee was limited, with tenderness on palpation over the medial joint line. Blood parameters showed an elevation of CRP of 10.9mg/dL (normal range less than 0.3 mg/dL). Plain radiographs of the right knee show in-situ right knee arthroplasty implants with no evidence of loosening (Figure 6). The patient was worked up for right knee PJI (Tsukayama III) and subsequently underwent DAIR with the change of PE and insertion of calcium sulfate beads (antibiotic stimulan) after three days of admission. Methylene blue staining showed extension into the right suprapatellar pouch medially and posteriorly. Intraoperatively, examination of the femoral and tibial components showed no loosening. Adequate debridement was done so that all methylene blue-stained tissue was excised. In this instance, 1 g vancomycin mixed with bone stimulan was inserted prior to wound closure (Figure 7). Post-operative IV cefazolin was administered for four weeks duration before changing to oral Rifampicin and Bactrim for another six weeks (10 weeks duration of antibiotics). Post-operative repeated X-ray as shown (Figure 8). Blood parameters showed improvement with the decrement of CRP to 0.26 mg/dL (Table 3). The surgical wound over the right knee, reviewed during clinic follow-up, shows a healed scar with good function. Blood parameters were normalized during the six-month follow-up.

**Table 3:** C- reactive protein (CRP) and white blood cell (WBC) trend

Date	CRP (<0.5 mg/dL)	WBC (4-10x10^9/L)
12/9/2022	10.9	14.4
20/9/2022	2.4	13.9
28/9/2022	3.8	9.7
16/10/2022	0.9	6.1
30/10/2022	0.1	9.4
28/11/2022	0.26	5.6

## Discussion

In this case series, we present three instances of PJI of total knee replacements successfully managed with DAIR, accompanied by the insertion of vancomycin beads and/or calcium sulfate beads. All three cases show promising results of wound closure with normalized infectious parameters. The patient is also currently asymptomatic and not complaining of knee pain over the respective operative site. The successful outcome in all three cases can be attributed to several critical factors.

First, early intervention and meticulous surgical debridement played pivotal roles in eradicating the infection [5]. This approach is supported by literature indicating that prompt and thorough debridement significantly enhances the success rate of DAIR procedures. Timely intervention is crucial in reducing the spread of infection, preserving the integrity of the implant, and preventing systemic complications. A recent meta-analysis by Gramlich et al. (2020) revealed that early debridement within a specific time frame after PJI diagnosis significantly increased the success rates of DAIR [6]. The study underscored the importance of prompt surgical intervention in the management of PJIs to enhance the chances of successful implant retention. Methylene blue is a cationic dye that can bind to eukaryotic cells and bacterial biofilms. Upon binding, the dye will stain these structures and can be a useful tool in guiding the debridement of biofilms. In vitro models have demonstrated successful staining of *Staphylococcus epidermidis* biofilms on orthopedic implants [7]. More recently, a study on the clinical application of methylene blue has demonstrated improved identification of staphylococcal biofilms in a comparison of stained versus unstained tissue in cases of PJI [8]. In this study, methylene blue was diluted to a concentration of 0.1% and instilled for 60 seconds over the surgical site after the removal of the PE. The usage of methylene blue as an adjuvant in the management of PJIs has garnered attention due to its ability to enhance bacterial killing and biofilm disruption. Recent research by Shi et al. (2021) demonstrated the efficacy of methylene blue in combination with antibiotics for the eradication of biofilms and the improvement of antimicrobial activity [9]. The study suggested that the incorporation of methylene blue in the treatment protocol of DAIR could contribute to higher success rates in eradicating infections and preventing biofilm formation on prosthetic joints.

Second, the use of vancomycin beads provided a high local concentration of antibiotics, which is essential for tackling biofilm-associated bacteria, a common challenge in PJI. The efficacy of vancomycin beads in conjunction with DAIR has been documented in the literature [10] and was reflected in the positive outcomes of our cases. The local delivery of antibiotics, such as vancomycin, through the usage of antibiotic-loaded beads, has been recognized as an effective strategy to achieve high concentrations of antimicrobials at the infection site while minimizing systemic exposure. Recent work by Smith et al. (2019) outlined the benefits of vancomycin beads in reducing bacterial biofilm formation and enhancing the local delivery of antibiotics in PJIs [11]. The study supported the use of vancomycin beads as an adjunct to DAIR, emphasizing its role in improving the success rates of implant retention in PJIs.

The functional outcomes for the patients were remarkable, with all three returning to their pre-infection level of activity and reporting satisfactory pain control. These results are consistent with findings from other studies [5] that have reported improved outcomes with the DAIR approach when combined with adjuvant therapies. While our results are promising, it is important to consider the limitations inherent in the DAIR procedure. Patient selection, the timing of intervention, and the nature of the infecting organism are all factors that can influence the success of the treatment. Our series adds to the growing body of evidence supporting the use of DAIR with vancomycin beads in appropriately selected patients, but further research is needed to solidify these findings and explore the long-term outcomes of such interventions.

## Conclusions

The DAIR method has been identified as a potentially effective therapeutic approach for PJI in TKA. The utilization of this treatment modality has demonstrated a high degree of efficacy in the complete elimination of infection, leading to enhanced functional outcomes and a decreased necessity for more invasive surgical interventions that carry a higher risk of adverse health outcomes. The success of DAIR in PJIs is influenced by several factors, including meticulous debridement, the usage of methylene blue, early debridement, and the usage of vancomycin beads for local antibiotic delivery. These factors play a crucial role in optimizing the effectiveness of DAIR and improving the outcomes for patients with PJIs. The references provided from recent journal articles in orthopedics highlight the significance of these factors in contributing to the success of DAIR, thereby offering valuable insights for the management of PJIs in orthopedic practice.
